# The evolution of the major histocompatibility complex in upstream versus downstream river populations of the longnose dace

**DOI:** 10.1002/ece3.2839

**Published:** 2017-04-01

**Authors:** Erika Crispo, Haley R. Tunna, Noreen Hussain, Silvia S. Rodriguez, Scott A. Pavey, Leland J. Jackson, Sean M. Rogers

**Affiliations:** ^1^ Department of Biological SciencesUniversity of CalgaryCalgaryABCanada; ^2^ Department of BiologyPace UniversityNew YorkNYUSA; ^3^ University of New Brunswick Saint John & Canadian Rivers InstituteSaint JohnNBCanada; ^4^ Present address: Touro College of PharmacyNew YorkNYUSA; ^5^ Present address: Developmental BiologySloan‐Kettering InstituteNew YorkNYUSA

**Keywords:** diversifying selection, heterozygote advantage, major histocompatibility complex, negative frequency‐dependent selection, next‐generation sequencing, overdominance

## Abstract

Populations in upstream versus downstream river locations can be exposed to vastly different environmental and ecological conditions and can thus harbor different genetic resources due to selection and neutral processes. An interesting question is how upstream–downstream directionality in rivers affects the evolution of immune response genes. We used next‐generation amplicon sequencing to identify eight alleles of the major histocompatibility complex (MHC) class II β exon 2 in the cyprinid longnose dace (*Rhinichthys cataractae*) from three rivers in Alberta, upstream and downstream of municipal and agricultural areas along contaminant gradients. We used these data to test for directional and balancing selection on the MHC. We also genotyped microsatellite loci to examine neutral population processes in this system. We found evidence for balancing selection on the MHC in the form of increased nonsynonymous variation relative to neutral expectations, and selection occurred at more amino acid residues upstream than downstream in two rivers. We found this pattern despite no population structure or isolation by distance, based on microsatellite data, at these sites. Overall, our results suggest that MHC evolution is driven by upstream–downstream directionality in fish inhabiting this system.

## 1 Introduction

Upstream versus downstream river habitats have been shown to harbor divergent biological resources. A classic example includes Trinidadian rivers, in which piscivorous fishes are found downstream but not upstream of waterfalls, and downstream guppies (*Poecilia reticulata*) have evolved predator‐evasion phenotypes distinct from upstream counterparts (e.g., Endler, 1980; Magurran, Seghers, Carvalho, & Shaw, 1992; O'Steen, Cullum, & Bennett, 2002). In particular, downstream areas have been shown to typically harbor increased intraspecific genetic diversity relative to populations upstream in the same rivers (review: Paz‐Vinas, Loot, Stevens, & Blanchet, 2015). This pattern was shown to be particularly strong in organisms without overland dispersal, such as fishes, and was not associated with any other particular species traits (Paz‐Vinas et al., 2015). Reasons for this upstream–downstream directionality could be downstream‐biased dispersal, founder effects upstream, and increased habitat diversity downstream (Paz‐Vinas et al., 2015). An additional possibility is that downstream and upstream sites differ ecologically such that divergent selection acts between upstream and downstream populations, leading to adaptive upstream–downstream divergence (e.g., as in guppies, above).

Globally, rivers are contaminated by municipal/industrial wastewaters and agricultural runoff, and contaminants are most concentrated downstream of municipalities and agricultural areas (Evans, Jackson, Habibi, & Ikonomou, 2012; Jeffries, Jackson, Ikonomou, & Habibi, 2010; Quinn, Rasmussen, & Hontela, 2010). Numerous studies have demonstrated direct effects of environmental contaminant exposure to field‐based fish populations. For example, contaminants with endocrine‐disrupting properties negatively impact populations of a small cyprinid (longnose dace, *Rhinichthys cataractae* Valenciennes 1842). Populations of longnose dace exposed to contaminants have female‐biased sex ratios (Jeffries, Nelson, Jackson, & Habibi, 2008; Jeffries et al., 2010), and many individuals have intersex gonads (Evans et al., 2012; Tunna, 2014). Additionally, the expression of vitellogenin (egg yolk precursor protein) is higher in fish located downstream of some municipal areas (Evans et al., 2012; Jeffries et al., 2008). Such observations reveal the direct effects that contaminants may have on freshwater fishes.

Contaminants may also indirectly impact fish by affecting host–pathogen interactions. Two hypotheses exist for the effects of environmental stress on infectious disease in fishes. First, environmental contaminants may negatively impact pathogens, such that fish from contaminated waters have reduced pathogen loads (Gheorghiu, Cable, Marcogliese, & Scott, 2007; Lafferty & Kuris, 1999). This would be the case if contaminants reduce the density of intermediate hosts or if pollutants, such as heavy metals, are more toxic to pathogens than to hosts (review: Lafferty & Kuris, 1999). Second, environmental contaminants may immunosuppress fish so that they are more susceptible to infectious disease in general (Cohen, Tirindelli, Gomez‐Chiarri, & Nacci, 2006; Lafferty & Kuris, 1999; Marcogliese & Pietrock, 2011; Snieszko, 1974; Valtonen, Holmes, & Koskivaara, 1997). These two hypotheses are not mutually exclusive. Conceivably, the first hypothesis might be correct for certain pathogen species, whereas the second hypothesis might be *generally* correct, or correct in association with other pathogen species. Given that contaminants vary between upstream–downstream river locations, and as they may impact pathogen communities, it is interesting to study the evolution of immune response genes between upstream–downstream localities.

The major histocompatibility complex (MHC) is a set of pathogen‐specific molecules in vertebrates that bind antigens and presents them to T cells. The MHC is an important component of the vertebrate immune system and targets extracellular (MHC class II) and intracellular (MHC class I) pathogens (review: Sommer, 2005). MHC molecules typically show extraordinary intraspecific and intra‐individual diversity due to the presence of multiple paralogous genes, and balancing selection acts to increase allelic and nucleotide diversity of MHC genes (reviews: Piertney & Oliver, 2006; Sommer, 2005). MHC diversity has also been implicated in mate choice, whereby individuals choose their mates based on some intrinsic ability to detect which MHC alleles are compatible with their own, which can in turn influence MHC diversity (Kamiya, O'Dwyer, Westerdahl, Senior, & Nakagawa, 2014; Milinski, 2006). Several studies in vertebrates, including fish, have shown cases of balancing and directional selection on MHC genes in populations from different environments (reviews: Eizaguirre & Lenz, 2010; Neff, Garner, & Pitcher, 2011; Piertney & Oliver, 2006). Balancing selection refers to pressure imposed by a diverse pathogen community that leads to an increase in allelic, genotypic, or sequence variation within a host population over evolutionary time (Fijarczyk & Babik, 2015; Hedrick, 1999). One type of balancing selection is negative frequency‐dependent selection, which occurs when rare alleles or genotypes are favored. Another type of balancing selection is heterozygote advantage, which occurs when heterozygotes have higher fitness than homozygotes. Directional selection, on the other hand, refers to selection that acts on a particular allele to increase its frequency in a population. For example, killifish (*Fundulus heteroclitus*) from estuaries and marshes with varying levels of PCB contamination have exhibited patterns of both types of selection on the antigen‐binding region of the MHC class II β subunit in populations from contaminated sites (Cohen, 2002). That study used populations that were isolated from each other, and thus, different gene frequencies could have been due to drift or different pathogen exposures. Balancing selection tends to reduce population differentiation because there is an inverse relationship between allelic diversity and genetic divergence, whereas divergent selection would cause the opposite pattern.

**Figure 1:**
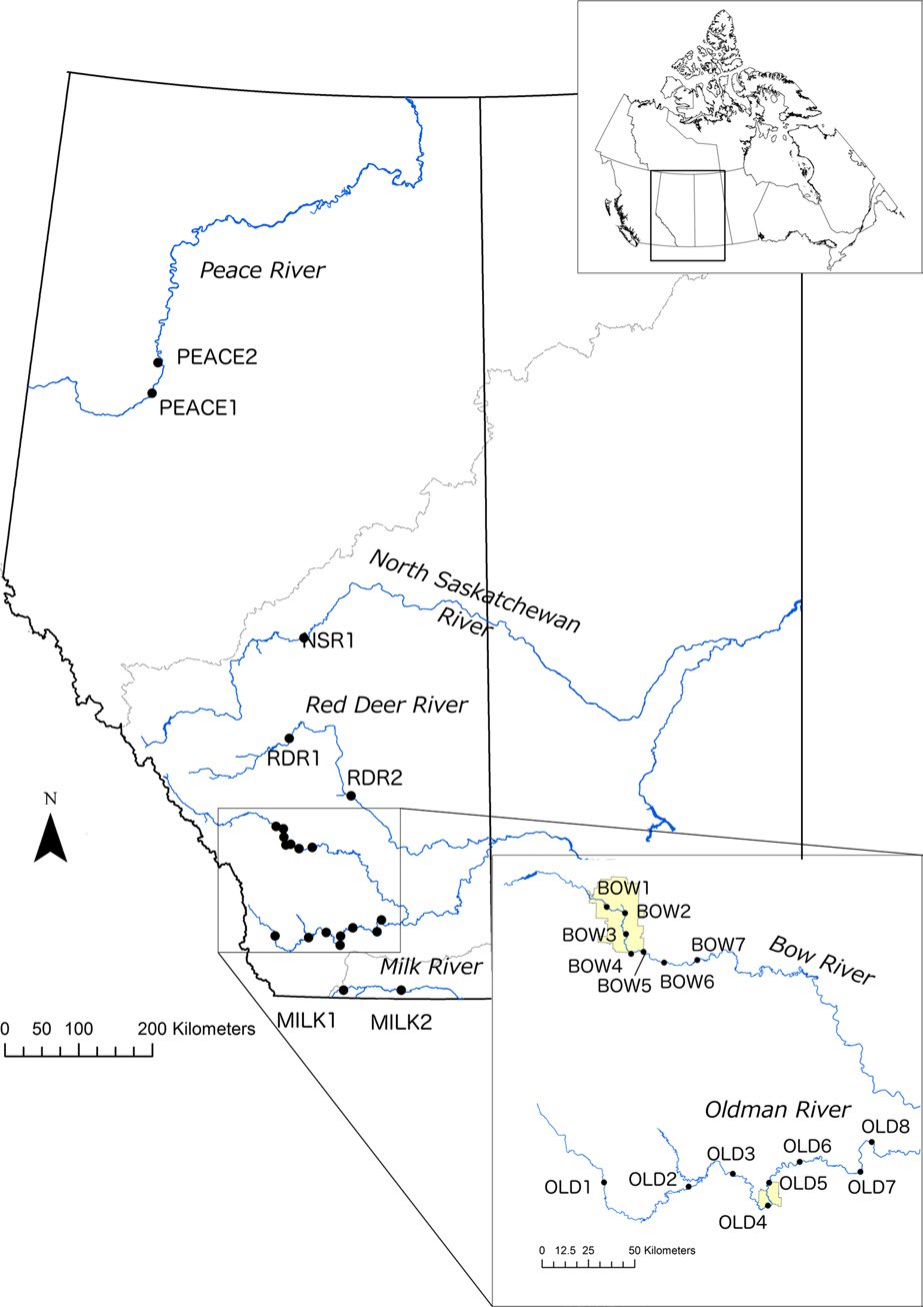
Map of sampling locations. Samples from all sites were used for microsatellite analysis. Samples from BOW1, BOW7, OLD1, OLD8, MILK1, and MILK2 were used for MHC analysis

We used next‐generation sequencing to sequence nucleotide variation in the MHC class II β subunit in longnose dace populations occurring upstream and downstream of municipal and agricultural areas in three headwater rivers of the South Saskatchewan River Basin in southern Alberta, Canada (Figure 1). Cyprinid species have been found to have either one (DAB3 or DAB3‐like; e.g., Šimková, Civáňová, Gettová, & Gilles, 2013) or two (DAB1/2 and DAB3/4; e.g., Osborne & Turner, 2011) paralogs of the MHC II β. We used alleles from this subunit of the MHC, particularly the second exon, because of its known involvement in peptide binding, and primers have already been designed for their amplification in longnose dace (Girard & Angers, 2011). We contrast these patterns of MHC variation with patterns of population structure observed using putatively neutral microsatellite loci. If parallel patterns of diversity in microsatellite and MHC alleles are observed, we cannot rule out the possibility that MHC variation is driven by neutral processes (gene flow and drift). However, if different patterns are observed between marker types, stronger evidence will be provided for selection's role on MHC variation.

We performed a set of analyses at the genotypic (allele frequency) and nucleotide sequence levels to measure MHC variation among and within individuals, populations, and rivers. MHC divergence between upstream–downstream populations, in the absence of significant microsatellite variation, would provide evidence that directional selection is occurring. MHC variation that is significantly higher in some sites than in others within rivers, in the absence of increased microsatellite variation, would suggest that balancing selection is occurring within the populations in those sites. Unidirectional water flow can shape population structure by promoting biased gene flow from upstream to downstream (Morrissey & de Kerckhove, 2009), which can increase diversity downstream relative to upstream. Thus, we contrast patterns of diversity between MHC and microsatellite markers to infer a role of selection versus neutral processes in shaping genetic variation.

## 2 Materials and Methods

### 2.1 Sampling protocol and site descriptions

Longnose dace were sampled from the Arctic, Hudson Bay, and Gulf Coast drainages in Alberta, Canada (Figure 1). These drainages have been separated from each other for at least 10,000 years (McPhail & Lindsey, 1970). Two sites were sampled in the Peace and Milk rivers, which are part of Arctic and Gulf of Mexico drainages, respectively (Figure 1). Four headwaters of the Hudson Bay drainage were sampled, including the North Saskatchewan, Red Deer, Bow, and Oldman rivers (Figure 1). The Bow River is the source water to the City of Calgary (51°3′0″N, 114°4′0″W; population ~1,300,000) and has 13 water conveyance structures, but only three occurring between the sampling sites (Figure 1). The Oldman River is source water for the City of Lethbridge (49°41′39″N, 112°49′58″W; population ~90,000) and flows through intensive agricultural land (Oldman Watershed Council, 2005). There is one dam (Oldman Dam) within the sampling area. Other sites sampled within the Saskatchewan River drainage included one site on the North Saskatchewan River upstream of the City of Edmonton (53°32′36″N, 113°29′26″W; population ~800,000), and two sites on the Red Deer River. Coordinates for all sampled sites are available in Table S1. The Red Deer River has no identified potential barriers to gene flow between sites we sampled. There are two water conveyance structures on the South Saskatchewan River downstream before it merges with the North Saskatchewan River to form the Saskatchewan River. All sites were at least 5 km apart by river distance, which is substantially larger than the <100 m estimated home range length of longnose dace (Hill & Grossman, 1987).

At each site, between 21 and 50 longnose dace were sampled by backpack electrofishing (Smith‐Root Model 12‐B POW) during late summer (August–September) in 2010 and 2012, and May 2013. During 2010 and 2012, mature fish (>45 mm) were sacrificed with either MS‐222 (Sigma Aldrich), a quick cut to the spinal cord with scissors, or 250 ppm clove oil (eugenol) in accordance with Canadian Council on Animal Care (CCAC) standards. During 2013, fin clips were sampled from fish of all sizes and individuals were released at the location from which they were collected. All tissues were preserved in 95% ethanol.

In the Oldman River, natural and synthetic hormones and veterinary pharmaceuticals (17β‐estradiol, estriol, estrone, mestranol, α‐zearalanol, and 19‐norethindrone) have been found in downstream sites, but not the upstream site (Evans et al., 2012; Jeffries et al., 2010). Other chemicals found in higher concentrations downstream of Lethbridge relative to the upstream Olin Bridge site included cholesterol and cholesterol derivatives, coprostan‐3‐one, ergosterol, and several phytosterols (Evans et al., 2012). Only testosterone and bisphenol A were not appreciably greater in concentration downstream relative to upstream in the Oldman River (Evans et al., 2012). Similar comparisons are not available for the Bow River.

Other physical and chemical attributes do not differ greatly between upstream and downstream localities in the Bow and Oldman rivers, based on 10 years of data collected by Alberta Environment and Parks at monitoring stations (Table 1). In the Bow River, numerous hypolimnetic withdrawal dams and weirs affect particle transport; therefore, turbidity, total nitrogen, and total phosphorus differ greatly between upstream and downstream locations. Data collected upstream in the Bow River were at a site located 18 km downstream of Ghost Reservoir, which may account for lower temperature, turbidity, and total phosphorus and nitrogen in comparison with the downstream site. Irrespective of upstream–downstream directionality, the Bow and Oldman rivers are characterized by differences in temperature due to differences in latitude. Intensive canola and barley production occurs in the Bow River downstream of Calgary, and there are approximately 4,500 feedlots in southern Alberta, many of which occur along or in tributaries of the Oldman River (LJJ, personal observations). Additionally, the sizes of urban areas differ between the two rivers, with 468,000,000 L/day of wastewater discharged from Calgary into the Bow River, and 35,000,000 L/day from Lethbridge into the Oldman River. Condition factors and gonadosomatic indices differed slightly between upstream–downstream sites and between rivers in longnose dace collected in spring 2005 for a different study, suggesting different selective pressures (Table 1).

**Table 1:** Characterization of upstream and downstream sites within the Bow and Oldman rivers, obtained from Alberta Environment and Parks (http://www.environment.alberta.ca/apps/basins/default.aspx). Data are averages, with standard errors in parentheses. TP is the total phosphorus, and TN is the total nitrogen (sum of ${\text{NO}}_{3}^{-}$ + ${\text{NO}}_{2}^{-}$ + NH_3_). CF is condition factor, and GSI is gonadosomatic index, of longnose dace collected in spring 2005 (not used in the present study). The fish from upstream in the Bow River used for the CF and GSI measures were from Edworthy rather than from the site indicated here for which water quality metrics were obtained

River	Site	Temp. (°C)	pH	Turbidity (NTU)	Conductivity (μS/cm)	TP (μg/L)	TN (μg/L)	Male CF	Male GSI	Female CF	Female GSI
Bow	51°10′57″; 114°29′13″ (upstream)	6.3 (0.6)	8.20 (0.02)	3.5 (1.0)	287.2 (3.0)	5 (1)	90 (5)	9.64 (0.13)	1.06 (0.11)	9.02 (0.13)	4.68 (0.25)
Bow	50°49′50″; 113°25′09″ (downstream)	7.0 (0.7)	7.98 (0.04)	16.1 (4.8)	359.7 (4.8)	57 (5)	1014 (57)	10.51 (0.14)	1.55 (0.14)	10.07 (0.15)	4.51 (0.51)
Oldman	49°47′24″; 113°07′24″ (upstream)	9.7 (0.7)	8.17 (0.02)	54.4 (16.2)	330.6 (4.5)	78 (27)	124 (18)	8.36 (0.18)	0.68 (0.07)	8.74 (0.09)	4.34 (0.33)
Oldman	49°57′40″; 112°05′05″ (downstream)	9.8 (0.7)	8.31 (0.03)	36.6 (8.6)	363.3 (4.9)	85 (22)	165 (24)	9.35 (0.23)	1.14 (0.11)	9.23 (0.10)	6.11 (0.21)

### 2.2 MHC genotyping

Longnose dace used for the MHC analyses were sampled in 2012 at six sites in southern Alberta. These sites included Edworthy Park (upstream; BOW1 in Figure 1) and Carseland Weir (downstream; BOW7) in the Bow River, Olin Bridge (upstream; OLD1) and Highway 36 (downstream; OLD8) in the Oldman River, Weir Bridge (downstream; MILK2) in the Milk River, and Highway 62 (MILK1) in the North Fork of the Milk River (Figure 1). The two sites in the Bow River are 88 km apart, and the two sites in the Oldman River are 167 km apart.

DNA from 20 fish per site was extracted using Qiagen DNeasy Blood and Tissue Kit spin columns and eluted in PCR‐grade water. We created amplicon libraries for the second exon of the MHC II β subunit, using primers that amplified a 236‐bp segment (207 bp on the exon and 29 bp on the intron between the second and third exons), using the forward primer 5′‐CCAGTGACTACAGTGATATGG‐3′ and the reverse primer 5′‐AGTGGACACACATGGTGAG‐3′ (from Girard & Angers, 2011). This segment was chosen for its known peptide‐binding properties (Girard & Angers, 2011) and high variability in cyprinids (e.g., Ottová et al., 2005). Roche 454 fusion primers were used, which included the Lib‐L adaptors, key, multiplex identifiers (forward primer only), and the primer sequence. We amplified the DNA using the following conditions: 94°C for 1 min; 25–35 cycles of 94°C for 45 s, 54°C for 30–45 s, 72°C for 30–90 s; followed by 72°C for 10 min. PCR product was cleaned using a Qiagen MinElute Kit and quantified using the Take3 Micro‐Volume Plate in a BioTek Synergy H1 Hybrid Multi‐Mode Microplate Reader, using the machine's built‐in nucleic acid quantification program for the Take3 Plate. We then submitted 15 ng of each amplicon to Genome Quebec Innovation Centre for sequencing on the Roche 454 Junior (two runs) and FLX+ (one quarter run) using one‐way read technology. Twenty‐six individuals were amplified and sequenced twice to estimate an error rate for the library preparation, sequencing, and bioinformatic procedure. We obtained data for 95 individuals: 20 from Edworthy Park (BOW1), 17 from Carseland Weir (BOW7), 20 from Olin Bridge (OLD1), 19 from Highway 36 (OLD8), 18 from Weir Bridge (MILK2), and 1 from Highway 62 (MILK1) (Figure 1). Sample sizes lower than 20 per site were due to amplification failure (Highway 62) or sequencing failure.

Data were filtered and alleles were identified using the bioinformatic procedure of Pavey et al. (2013; see also Lamaze et al., 2014). The first step aligns reads within each sample, calculates the pairwise distance matrix, and uses hierarchical clustering to identify putative alleles within samples. The minimum internal branch length was set to 0.1. The second step aligns all putative allele sequences, clustering sequences based on identity, and defining global consensus alleles based on clusters with at least two identical sequences. For step three, the original data were subjected to a BLAST search against the consensus alleles; an allele was assigned to an individual if it comprised at least 20% of the reads in the data file for a given sample. Initially, only putative alleles that were present in at least two samples were further considered. We then repeated the entire procedure, allowing putative alleles that were present in only one sample to be considered, so as to not bias our data set against rare alleles. These two alternative approaches yielded identical genotyping results. These procedures identified a total of eight alleles with a maximum of two alleles per sample, indicating that we amplified a single locus. Only one individual amplified for the North Milk River population, so we combined that individual with the other Milk River samples for data analyses.

### 2.3 Microsatellite genotyping

DNA from all sampled fish was isolated using a standard phenol chloroform and ethanol precipitation protocol (Sambrook & Russell, 2001). A total of 12 microsatellite loci were amplified, including 11 dinucleotide repeat loci and one tetra‐nucleotide repeat locus. Four primer pairs were characterized and optimized from Girard and Angers (2006; Rhca16, Rhca20, Rhca23, and Rhca24) and four from Turner, Dowling, Broughton, and Gold (2004; Lco1, Lco3, Lco4, and Lco8). An additional four primer pairs were developed with shotgun 454 pyrosequencing (Tunna, 2014; Rhca203, Rhca211, Rhca225, and Rhca249). We prepared 500 ng/μL of genomic DNA for a 1/16 of a run on a Roche 454 platform (Plant Biotechnology Institute). Shotgun sequencing resulted in 65,040 reads with a mean read length of 420 bp. We used sff_extract to convert sff to fasta file format (Blanca & Chevreux, 2012) and removed adaptors with the FASTX‐Toolkit (Gordon, 2010). We identified microsatellite repeat regions (di‐, tri‐, tetra‐) in MSATCOMMANDER v. 1.0.8 and designed primers with the Primer3 (Rozen & Skaletsky, 2000) algorithm implemented in MSATCOMMANDER v. 1.0.8 (Faircloth, 2011). This procedure identified 75 possible primer pairs resulted, of which only four amplified and were polymorphic on up to 48 individuals. PCR amplification of primers designed in this study was carried out in 10 μL reaction volumes containing 1× reaction buffer, 2 mmol/L MgSO4, 0.1 mmol/L of each dNTP, 0.2 μmol/L of each F and R primer, 0.5 mmol/L of BSA, 0.1 U of *Taq* polymerase (New England Biolabs), and 4 ng/μL of template DNA (thermal cycler conditions provided in Tunna, 2014). Electrophoresis consisted of pooling PCR products with an internal size standard (LIZ 500 bp; Applied Biosystems) on the Applied Biosystems 3500xL Automated Sequencer. Allelic sizes (in base pairs) were determined by reference to the internal sizing standard in the software GENEMAPPER v. 3.7 (Applied Biosystems).

### 2.4 MHC data analysis

Of our 26 samples that were amplified and sequenced twice, only one (from Edworthy Park, BOW1) was assigned a different genotype in each of two replicates; thus, our estimated repeatability rate was 96.2% across the library preparation, sequencing, and bioinformatic procedure. This rate is higher than that from other studies that used a similar approach to estimate repeatability (e.g., 87.1% in Herdegen, Babik, & Radwan, 2014; 81.5% in Rico et al., 2016). We excluded that one sample from subsequent analyses. Data for 94 individuals remained. To visually explore the variation among the eight alleles, we constructed a neighbor‐joining tree using MEGA v. 6.06 (Tamura, Stecher, Peterson, Filipski, & Kumar, 2013). We used the Jukes–Cantor model, assuming uniform rates among sites.

We included two categories of analyses to test our hypotheses: those that tested for variation *among* populations (divergent selection or drift) and those that tested for variation *within* populations and individuals (balancing selection). Within each category, we included analyses that were based on allele frequencies (genotypes) and nucleotide variation. Genotype‐based analyses have the benefit of identifying variation at the individual level, but exclude information on nucleotide sequence variation among alleles.

We estimated pairwise population divergence as Φ_ST_, with significance levels based on 110 permutations, using Arlequin v. 3.5.1.2 (Excoffier & Lischer, 2010). We estimated the average number of nucleotide substitutions per site between populations (Dxy) using DnaSP v. 5.10.01 (Librado & Rozas, 2009). We used analysis of molecular variance (AMOVA) to address the hypothesis relating to divergence among populations and the hypothesis relating to variation within populations. To include variation at the individual level, we used genotypic data for this analysis. The AMOVA was conducted using Arlequin and was based on a matrix of pairwise differences, with significance levels based on 3,024 permutations. We estimated variation within individuals, among individuals within populations, between populations within rivers, and among rivers. To test for balancing selection within individuals, we calculated observed heterozygosities and estimated expected heterozygosities, using Arlequin. This analysis used a Markov chain to perform an exact test of the Hardy–Weinberg equation, with the chain consisting of 100,000 steps and 1,000 dememorization steps. We also conducted the Ewens–Watterson neutrality test in Arlequin, with 1,000 simulated samples.

We estimated population descriptive statistics based on nucleotide variation, including the number of haplotypes, haplotype diversity, the number of polymorphic sites, the average number of nucleotide differences, and nucleotide diversity using DnaSP. For each population, we used DnaSP to estimate the number of synonymous and nonsynonymous sites, and the number of synonymous and nonsynonymous mutations, and calculated the dN/dS ratio. We then used MEGA to conduct a codon‐based *Z*‐test of historical neutral evolution for each population, using the Nei–Gojobori method (Nei & Gojobori, 1986), with significance levels based on 500 bootstrapped replicates. This test is used to detect dN‐dS estimates that are significantly different from zero (i.e., dN ≠ dS). Analyses comparing synonymous to nonsynonymous substitutions were performed for the coding region only.

To account for recombination within the coding region, we used OmegaMap v. 0.5 (Wilson & McVean, 2006) to simultaneously estimate dN/dS and recombination rate, for each population separately, using a Bayesian framework. In our parameter file, the equilibrium frequency of each codon was set to 1/61 and the number or orderings was set to 10, with a list of random orderings generated using the program included in OmegaMap. μ and κ priors were set to improper inverse; ω (dN/dS) and ρ (recombination rate) priors were set to inverse, which corresponds to a log scale uniform distribution. The ω and ρ models were set to variable, which is a block‐like model that allows adjacent sites to share the same recombination rate; the block length was set to 10. Although it would have been desirable to allow amino acid residues to vary independently, we were not able to attain MCMC convergence, even with a very high number of iterations, when we selected “independent” for the ω model, or when we selected smaller block sizes. For each population, we ran two independent analyses with different starting values for μ, κ, and indel; the number of iterations was set to 500,000 for one run and 1 million for the second run, with every 100th iteration written to the output file. We also ran one analysis, using these parameters, on the entire data set. We used Tracer v. 1.5 (Rambaut, Suchard, Xie, & Drummond, 2014) to ensure that the run time was sufficient (i.e., effective sample sizes >200).

### 2.5 Microsatellite data analysis

To test Hardy–Weinberg and linkage equilibrium at each locus, we used GENEPOP v. 4.1.4 (Raymond & Rousset, 1995; Rousset, 2008). Type I error rates for multiple comparisons were adjusted using a false discovery rate procedure (Benjamini & Yekutieli, 2001) and sequential Bonferroni method (Holm, 1979) as implemented in the *multtest* package in R v. 3.0.3 (R Core Team, 2014 ). To test whether null alleles were present at any loci, we used FreeNA (Chapuis & Estoup, 2007; Dempster, Laird, & Rubin, 1977). One locus, Rhca203, did not meet Hardy–Weinberg or linkage equilibrium expectations and exhibited significant evidence of null alleles (data not shown) and was eliminated from analyses. Estimates of rarified allelic richness (*A*_R_) and expected heterozygosity (*H*_E_) were estimated in FSTAT v. 2.9.3.2 (Goudet, 1995). We used one‐way ANOVAs in R to test for variation in *A*_R_ and *H*_E_ among sites within each river (i.e., separate ANOVAs conducted for each river).

We used analyses of molecular variance (AMOVA), implemented in Arlequin v. 3.5.2.2, to test for hierarchal structure (Excoffier & Lischer, 2010). First, we tested for hierarchical structure among and within all six rivers (three hierarchical levels: rivers, sites within rivers, and within sites). Second, we tested for hierarchical structure within and between the Bow and Oldman rivers only, for which we sampled more densely and for which we also have MHC data. In both cases, we set the number of permutations to 3,000.

We conducted three separate tests for isolation by distance. River distance was measured as the shortest geographical distance (km) between sites connected by waterways (Google Earth v. 7.1.2.2014). We also tested isolation by distance using Euclidean distance (the shortest geographical distance) between population pairs, because populations may not have colonized via current waterways after the last glacial maxima. We used the *Geneclust* package (Ancelet, 2010) in R to estimate pairwise *F*_ST_ between populations (Weir & Cockerham, 1984). All geographical distances (Euclidean and river) were log‐transformed, and all genetic distances (*F*_ST_) were transformed as *F*_ST_ (1‐*F*_ST_). First, we conducted a Mantel test comparing pairwise genetic distance to Euclidean distances among all sampled sites. Second, we conducted partial Mantel tests comparing pairwise genetic distance to geographical distance along the river within the Bow River and within the Oldman River (two separate tests), while also controlling for the number of barriers (dams and weirs) separating sites. Mantel and partial Mantel tests were conducted with the *ecodist* package (Goslee & Urban, 2007) in R, with 100,000 permutations used to estimate significance levels for the correlation coefficients.

Finally, we used an individual‐based assignment approach as implemented in STRUCTURE v. 2.3.4 (Hubisz, Falush, Stephens, & Pritchard, 2009; Pritchard, Stephens, & Donnelly, 2000) to determine the number of genetic clusters (*K*) in the microsatellite data. We used the admixture model, and model parameters were set to default. Each simulation consisted of a burn‐in period of 100,000, and 10,000,000 Monte Carlo Markov Chain (MCMC) iterations after burn‐in to determine the probability of the model, as recommended by Gilbert et al. (2012). Simulations were run assuming *K* = 1 to 23 with genotypes from all locations. We also tested hierarchal structure by running simulations on samples collected from only the Bow and Oldman rivers (both rivers combined in the analysis) assuming *K* = 1 to 16. All simulations were replicated 20 times for each value of *K*. Simulations were run on a high‐performance computing cluster at the University of Calgary. Because the ∆*K* method cannot evaluate one genetic cluster (Evanno, Regnaut, & Goudet, 2005), we also used the mean Ln(P)*K* approach to determine the most likely number of genetic clusters. We used the “Greedy” algorithm in CLUMPP to combine replicate runs of *K* (Jakobsson & Rosenberg, 2007) and DISTRUCT to visualize population clusters (Rosenberg, 2004).

## 3 Results

### 3.1 MHC

**Figure 2:**
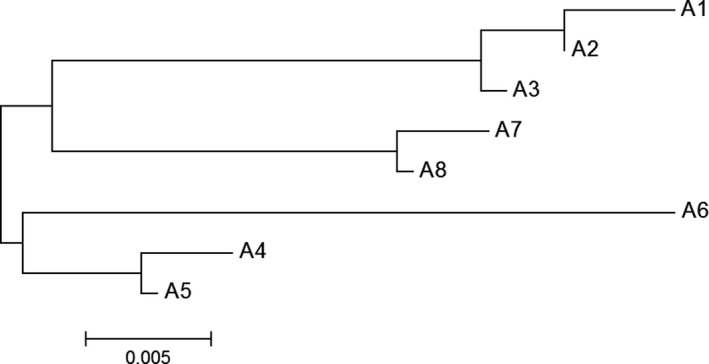
Neighbor‐joining gene tree for the eight identified MHC alleles (A1 through 8), constructed using MEGA6. The scale represents nucleotide substitutions per site

**Figure 3:**
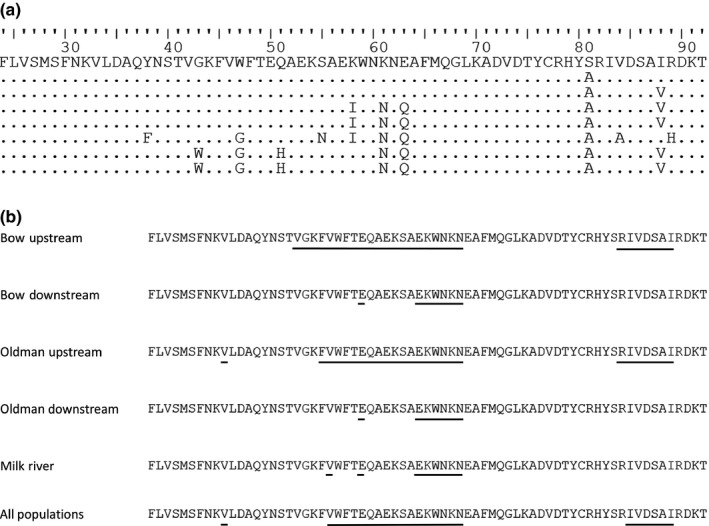
Amino acid sequences for part of the MHC IIβ exon 2 sequenced from longnose dace. (a) Amino acid sequences for each of the eight MHC alleles, corresponding to the eight alleles in Figure 2, starting with allele 1 at the top and proceeding in sequence to allele 8 at the bottom. (b) Amino acid residues (shown for allele A1) under diversifying selection (i.e., ω greater than expected under neutrality; posterior probability ≥.95 in two separate runs) in each population and across all populations, as inferred by OmegaMap, are underlined. Putative antigen‐biding sites, based on the crystalline protein structure from humans (Brown et al., 1993), are 28, 30, 32, 37, 38, 47, 56, 60, 61, 65, 68, 70, 71, 74, 81, 82, 85, 86, 88, and 89

The number of reads per amplicon ranged from 101 to 14,027; the average number of reads was 3,620 (±3,370; standard deviation). Our amplification and sequencing of part of the MHC II β gene yielded eight alleles (Figure 2; Tables 2 and 3), coding for a total of five different amino acid sequences (as determined using the translate tool in BioEdit v. 7.2.5; Hall, 1999; Figure 3a). These nucleotide and amino acid sequences were verified as belonging to the MHC II β exon 2 DAB3 locus using BLAST searches. In searches optimized for highly similar sequences, our sequences aligned to the MHC II β exon 2 of blacknose dace (*Rhinichthys atratulus*) and longnose dace (Girard & Angers, 2011), and the DAB3 locus of the MHC II β exon 2 of a hybrid between two European cyprinids, *Parachondrostoma toxostoma* and *Chondrostoma nasus* (Pctn‐DAB3*40 allele; Šimková et al., 2013), indicating that the DAB3 locus was probably analyzed in a previous study on longnose dace MHC variation (Girard & Angers, 2011; as in Osborne, Pilger, Lusk, & Turner, 2017). Nearly all alleles were found in every river with the exception of allele 5, which was only found in the Milk River (Table 2). (As all analyses except the AMOVA were conducted on a river‐by‐river basis, the anomalous allele from Milk River will not have impacted our conclusions about upstream–downstream comparisons within the Bow and Oldman rivers.) Alleles 5 and 6 were rare, present only twice and six times in the data set, respectively (Table 2). Allele 6 was the most distinct (Figure 2) and was only present at upstream sites (Table 2). There were 16 segregating sites, and the average number of nucleotide differences ranged from 4.52 to 5.59 among populations, while the nucleotide diversity ranged from 0.0192 to 0.0237 among populations (Table 3). The number of alleles was not greater downstream than upstream; a total of seven alleles were found in upstream sites, and a total of seven alleles were found in downstream sites, with six alleles found in both (Table 2).

**Table 2:** Number of each of the eight MHC alleles (as in Figure 2) in each population. Site codes are from Figure 1

Site code	Location	A1	A2	A3	A4	A5	A6	A7	A8	Total
BOW1	Edworthy Park (upstream)	3	9	10	6	1	2	–	7	38
BOW7	Carseland Weir (downstream)	2	7	11	6	–	–	–	8	34
OLD1	Olin Bridge (upstream)	1	11	8	7	–	4	–	9	40
OLD8	Highway 36 (downstream)	4	15	4	8	1	–	–	6	38
MILK1	Highway 62 (upstream)	–	1	–	1	–	–	–	–	2
MILK2	Weir Bridge (downstream)	1	2	12	15	–	–	5	1	36

**Table 3:** Descriptive statistics from DnaSP, and observed and expected heterozygosities from Arlequin. *n* is the number of sampled individuals. *h* is the number of MHC haplotypes. *H*_d_ is MHC haplotype diversity. *S* is the number of polymorphic sites. *k* is the average number of nucleotide differences. *P*_i_ is the nucleotide diversity. *H* is the heterozygosity (exp = expected, obs = observed). Asterisks indicate heterozygosities that differ significantly (*p* < .05)

River	Location	*n*	*h*	*H*_d_	*S*	*k*	*P*_i_	*H*_exp_	*H*_obs_
Bow	Up	19	7	0.82788	16	5.021	0.02128	0.841	0.737
Bow	Down	17	8	0.80493	12	4.522	0.01916	0.786	0.647
Oldman	Up	20	6	0.81282	16	5.591	0.02369	0.868*	0.500*
Oldman	Down	19	6	0.77240	12	4.573	0.01938	0.797	0.579
Milk		19	6	0.71693	12	4.578	0.01940	0.723	0.526

Pairwise Φ_ST_ was significant for population pairs that included the Milk River (Table S2). D_XY_ values ranged from 0.0191 (Bow and Oldman downstream populations) and 0.0227 (Oldman River upstream population and the Milk River; Table S2). The AMOVA revealed that while the majority of variation existed within individuals, this variation was less than expected (*s* ^2^ = 0.059, % variation = 62, *df* = 94, *p* = .009). On the other hand, more variation existed *within* sites than expected (*s* ^2^ = 0.041, % variation = 44, *df* = 89, *p* = .004), but there was no significant variation observed *among* sites (*s* ^2^ < 0, % variation < 0, *df* = 2, *p* = .705) or among rivers (*s* ^2^ < 0, % variation < 0, *df* = 2, *p* = .479). Thus, the unique allele found in the Milk River is not driving any significant divergence among sites or among rivers. Furthermore, observed heterozygosities were actually less than expected (although only significantly so for the Oldman River upstream site; Table 3), corroborating the AMOVA result that intra‐individual variation is less than expected. Furthermore, Ewens–Watterson's estimator indicated that homozygosity was greater than expected based on randomized samples at three sites (Oldman River upstream and both sites in the Bow River; one‐sided *p* < .05).

Another class of tests assumes that the rate of nonsynonymous and synonymous substitutions should be equal under neutrality. These tests estimate selection that occurred in the more distant past (Garrigan & Hedrick, 2003). The ratio of nonsynonymous to synonymous substitutions was >2.4 for all populations, although the *Z*‐test statistic (dN‐dS) was not significantly different from zero for any population (Table S3). We detected signatures of past recombination within our gene (DnaSP and OmegaMap, results not shown), and so the use of OmegaMap was appropriate for our data. When recombination has occurred, the false‐positive rate of detection of selection is inflated, because all nucleotides in a sequence do not share the same evolutionary history. OmegaMap uses an approximation to the coalescent that allows dN/dS to vary along the sequence (Wilson & McVean, 2006). OmegaMap results were nearly identical between two separate runs per population and inferred selection on individual amino acid residues. We considered an amino acid site to be under diversifying selection if the posterior probability of ω being greater than expected under neutrality was 0.95 or greater in both runs, when rounding to the nearest 100th of a decimal place. There were 68 amino acid residues in total that were coded for by the part of the exon that we sequenced. In each of the five populations, and across all populations, amino acid residues at positions 28 and 35–40 were under balancing selection (i.e., selection for increased nonsynonymous substitutions relative to neutral expectations; Figure 3b). In some populations, amino acids in positions 20–34 were also under balancing selection, and in the upstream populations in the Bow and Oldman rivers, amino acids in positions 60–66 were under balancing selection (Figure 3b). A larger sequence of amino acids was putatively under balancing selection in the two upstream populations than in the other three populations (Figure 3b). The only disagreement in balancing selection status between runs occurred at position 21 in the Oldman River upstream population, and at positions 24–27 in the Bow River downstream population. Across all populations, balancing selection was most similar to that of the Oldman upstream population, with the exception of two amino acid sites (Figure 3b).

### 3.2 Microsatellites

After removal of the Rhca203 locus and after sequential Bonferroni and false discovery rate correction, loci deviated from Hardy–Weinberg equilibrium in 22 tests, involving 13 sampling sites, and seven of these deviations were for the locus Rhca16 (Table 4). One pair of loci was in linkage disequilibrium at six sampling sites, and two pairs of loci were in linkage disequilibrium at one sampling site (Table 4). Genetic diversity (*H*_E_ and *A*_R_) was similar among sites and rivers, with one exception: Fish sampled from the Peace region had approximately half the diversity of that of all other sampling sites (Table 4). Diversity measures were comparable to recently developed microsatellite markers for longnose dace (Beasley, Lance, Ruskey, & Taylor, 2014). There was no significant difference in either allelic richness or gene diversity among sites within any of the five river systems in this study (Table S4).

**Table 4:** Number of individuals (*N*) and measures of genetic diversity for *Rhinichthys cataractae* sampled from 22 sites in three drainages in Alberta (Figure 1). *H*_o_ is mean observed heterozygosity across loci, *H*_E_ is mean expected heterozygosity across loci, and *A*_R_ is mean allelic richness. HWD identifies loci in Hardy–Weinberg disequilibrium after Bonferroni and false discovery rate correction. nLD is number of pairs of loci in linkage disequilibrium after Bonferroni and false discovery rate correction. BOW1 is the upstream and BOW7 is the downstream site sampled for the MHC. OLDMAN1 is the upstream and OLDMAN8 is the downstream site sampled for the MHC

Site	Year	*N*	*H*_O_	*H*_E_	*A*_R_	HWD	nLD
PEACE1	2012	48	0.32	0.31	4.39	Rhca225	–
PEACE2	2012	30	0.38	0.36	4.86	–	–
NSR1	2013	49	0.6	0.5	7.82	Rhca16, Lco1	–
RDR1	2013	21	0.63	0.59	7.91	–	–
RDR2	2013	45	0.70	0.65	7.91	Rhca24	–
BOW1	2010, 2012	91	0.70	0.63	8.20	Rhca16, Lco1, Lco3, Lco4	–
BOW2	2010	32	0.72	0.68	8.28	Rhca16	–
BOW3	2010	49	0.76	0.73	8.04	–	1
BOW4	2010	50	0.71	0.70	8.25	–	–
BOW5	2010	36	0.72	0.68	8.41	Rhca16	–
BOW6	2010	30	0.74	0.65	8.24	Rhca16, Lco4	–
BOW7	2010, 2012	97	0.69	0.61	8.12	Lco3, Lco4	1
OLDMAN1	2010, 2012	98	0.70	0.66	8.61	Rhca16, Lco4	–
OLDMAN2	2010	49	0.64	0.60	8.00	Rhca16, Rhca24	1
OLDMAN3	2010	49	0.71	0.66	8.4	–	1
OLDMAN4	2010	37	0.71	0.67	8.46	Lco1	–
OLDMAN5	2010	50	0.56	0.53	7.54	–	1
OLDMAN6	2010	52	0.63	0.58	7.74	Rhca16	–
OLDMAN7	2010	46	0.67	0.63	7.80	–	–
OLDMAN8	2010, 2012	100	0.68	0.60	8.24	–	2
MILK1	2013	50	0.71	0.66	9.02	Rhca24, Rhca211	1
MILK2	2013	25	0.70	0.65	9.09	–	–

Analyses of molecular variance detected significant population genetic structure in longnose dace among our six sampled rivers (*s* ^2^ = 0.14, % variation = 14, *df* = 5, *p* < .001). Additionally, we detected significant genetic population structure among sites within rivers (*s* ^2^ = 0.01, % variation = 1, *df* = 16, *p* < .001), with most of the genetic variation occurring within sites, although the variation within sites was significantly less than randomized values (*s* ^2^ = 0.89, % variation = 85, *df* = 1112, *p* < .001). In contrast to this result, no significant genetic structure occurred between the Bow and Oldman rivers when analyzed separately (*s* ^2^ < 0, % variation = 0, *df* = 1, *p* = .593). Sites within each river differed weakly but significantly in the test including only the Bow and Oldman rivers (*s* ^2^ = 0.01, % variation = 2, *df* = 13, *p* < .001). Most genetic variation occurred within rather than among sites, although again the randomized value was greater than the observed value (*s* ^2^ = 0.92, % variation = 98, *df* = 851, *p* < .001).

There was no evidence of isolation by distance in this system. For all sampled fish, *F*_ST_ did not correlate significantly with Euclidian distance (*r* = .060, *p* = .697). There was also no evidence of isolation by distance along the Bow River (partial Mantel *r* = .007, *p* = .977) or along the Oldman River (partial Mantel *r* = .374, *p* = .071), controlling for barriers.

**Figure 4:**
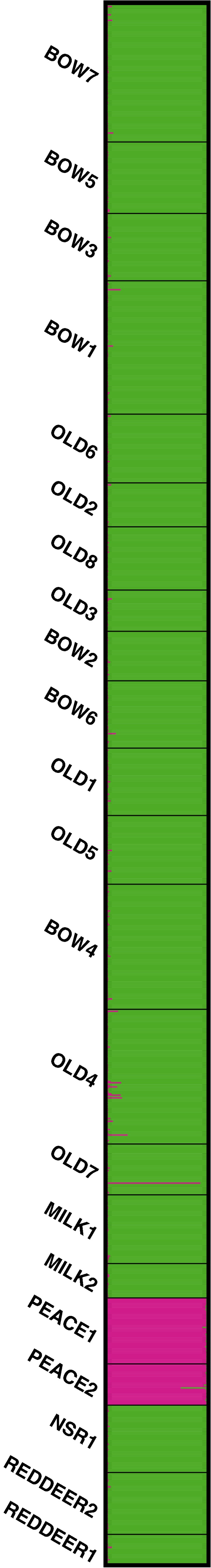
Population genetic structure of longnose dace from 22 sites in three drainages in Alberta. Each vertical line represents one individual, the colors represent genetic clusters, and the vertical distances are the relative assignment probabilities to the two genetic clusters. Visualized with DISTRUCT based on 20 independent runs, each using 100,000 burn‐in and 1,000,000 MCMC iterations

The Bayesian clustering algorithm STRUCTURE identified longnose dace from the Peace River system in northern Alberta as a separate genetic cluster from those sampled from the rest of Alberta, with two genetic clusters identified in the entire microsatellite data set (Figure 4). In the Bow and Oldman rivers, strongest support was provided for one genetic cluster.

## 4 Discussion

Overall, we found some evidence of balancing selection at the sequence level in the MHC II β exon 2 in longnose dace, but no evidence of heterozygote advantage or divergent selection. Balancing selection occurred at more amino acid residues at upstream versus downstream river sites. Analyses based on substitution rates tend to reflect more historical selection than do analyses based on Hardy–Weinberg equilibrium, because signals of selection at the nucleotide level may take long periods of time to erode in the absence of selection (Garrigan & Hedrick, 2003). Thus, it may be possible that selection was stronger in the past than in the present in our system, while still occurring within the time frame of the current distribution of the species along the Bow and Oldman rivers (given the parallel patterns of selection in these two rivers).

Although it is predicted that fish in riverine systems will show greater allelic diversity in downstream populations due to increased downstream dispersal and founder effects at upstream sites (Paz‐Vinas et al., 2015), we did not find this pattern in longnose dace for either MHC alleles or microsatellites. We detected low MHC haplotype diversity within sampled field populations, with only eight alleles identified, but high diversity among MHC haplotypes, with 16 polymorphic sites among these eight alleles. We identified a maximum of two MHC alleles per individual, suggesting that we amplified a single locus (DAB3). It is possible that paralogs exist, as in other cyprinid species (Ottová et al., 2005), but we were unable to amplify them with our primers. Low MHC variability corresponded with low microsatellite variability, with microsatellite allelic richness of ~8 in the Bow and Oldman rivers, and allelic richness ranging from 4 to 9 study‐wide, across 11 microsatellite loci (10 of which were dinucleotide repeat loci, one tetra‐nucleotide repeat locus). In a similar study on longnose dace from 27 rivers in Quebec, microsatellite allelic richness ranged from 2 to 5 in seven loci (six of which were dinucleotide repeat loci; Girard & Angers, 2006, 2011). Expected heterozygosities fell within the same range, 0.3–0.7 across populations, in both studies. Thus, the neutral diversity found in our study was comparable to that of other longnose dace populations.

Major histocompatibility complex II β exon 2 was more variable in our sampled populations than those from a previous study of longnose dace from 27 rivers in Quebec (Girard & Angers, 2011). This result was obtained despite the fact that neutral diversity was comparable between the two studies, as above. From 542 individuals, only four MHC alleles and a total of 11 polymorphic sites were identified in that study; it was likely that only DAB3 was analyzed in that study (Girard & Angers, 2011; Osborne et al., 2017). BLAST searches revealed that no alleles were identical between our study and the Quebec longnose dace study, with alleles from our study differing from between one and five nucleotide substitutions from alleles from the Quebec study. We can conclude that our MHC genotyping results were comparable to the previous study conducted on longnose dace from Quebec; low allelic and nucleotide diversity were observed in both systems, and allelic identities were similar even though the studies were conducted on opposite sides of the country in areas with different postglacial histories.

Other studies on cyprinids have shown strong positive selection on the second exon of the DAB3 locus (e.g., Collin, Burri, Comtesse, & Fumagalli, 2013; Ottová et al., 2005; Seifertová & Šimková, 2011), and whether DAB1 or DAB3 loci were more variable differed among species. In the North American Rio Grande silvery minnow (*Hybognathus amarus*), DAB1 had considerably more allelic diversity, with 55 alleles identified for DAB1 and only 13 alleles identified for DAB3 in a sample of 252 individuals (Osborne et al., 2017). Individuals each had ~1–2 alleles on average, with a maximum of four alleles in one individual (Osborne et al., 2017). In some European cyprinid species, however, DAB3 was the more variable locus. In European chub (*Squalius cephalus*), for example, 66 DAB3 and 45 DAB1 alleles were identified in a sample of 310 chub (Seifertová & Šimková, 2011). A higher number of DAB3 than DAB1 alleles were also found in native (*Parachondrostoma toxostoma*) and invasive (*Chondrostoma nasus*) cyprinids in southern France; 42 DAB3 and 13 DAB1 alleles were identified in the native species (119 individuals), and 23 DAB3 and 6 DAB1 alleles were identified in the invasive species (89 individuals) (Šimková et al., 2013). In a European minnow (*Phoxinus phoxinus*), DAB3 had greater amino acid diversity than DAB1 at the putative peptide‐binding region (Collin et al., 2013). In a study comparing 11 species of cyprinid (four to seven individuals per species), a comparable number of DAB1 and DAB3 alleles (40 and 42, respectively) were identified, with up to two DAB3 alleles per individual. Therefore, while the allelic variation we found is similar to the variability found in other studies on North American cyprinids, it is considerably less than that found at the DAB3 loci in European cyprinids. We see two possible biological reasons for the low genetic diversity observed in our system. Low genomewide diversity could be due to a recent postglacial expansion. The low variability in our samples, not only in the haplotype and sequence diversity of the MHC II β exon 2 alleles but also at microsatellite loci, coupled with our inability to detect isolation by distance using microsatellites, suggests that this is a plausible explanation. The Laurentide ice sheet covered the majority of North America, including Alberta, during the last glacial maxima, and thus longnose dace populations in our study would have been colonized via glacial refugia as the glaciers receded ~11,000 years ago (Shafer, Cullingham, Côté, & Coltman, 2010). Another, not mutually exclusive, possibility for low MHC haplotype and sequence diversity in longnose dace could be that other immune response genes or other MHC loci play a bigger role in their immune system, thus rendering low selection on the MHC II β. As noted above, many European cyprinid species have higher DAB3 than DAB1 diversity, but the reverse may have evolved in North American cyprinids. In Atlantic cod (*Gadus morhua*) and pipefish (*Syngnathus typhle*), as another example, MHC II genes have been lost, and increased diversity of MHC I genes in Atlantic cod appears to have assumed the function of the lost MHC II genes (Haase et al., 2013; Malmstrøm, Jentoft, Gergers, & Jakobsen, 2013; Star et al., 2011). Thus, any lack of diversity we identified may be limited to the MHC II β DAB3 locus and not to diversity of genes targeting extracellular pathogens in general.

No significant MHC variation existed among sites in an analysis of molecular variance, despite the fact that weak but significant differentiation occurred among sites at microsatellite loci. We can therefore conclude that divergent selection is playing no role in MHC II β variation among longnose dace populations in this region. This result contrasts studies on other fish populations; for example, a study on threespine stickleback found that of 329 identified MHC II β alleles, 143 were unique to a freshwater deme and 147 were unique to a saltwater deme. That study only examined two populations, however, so it is unclear whether these allelic differences were due to divergent selection between environments (McCairns et al. 2011). In sockeye salmon (*Oncorhynchus nerka*), significant MHC II β variation existed among beach, river, and stream ecotypes from multiple locations, and this variation was 30 times greater than variation at neutral SNP loci, suggesting divergent selection among ecotypes at this locus (Larson, Seeb, Dann, Schindler, & Seeb, 2014). In guppies, whether divergent selection influences MHC diversity between upstream–downstream populations, and whether this divergence was greater or less than divergence based on microsatellites, depends on the localities that were studied (Fraser, Ramnarine, & Neff, 2010; Herdegen et al., 2014; van Oosterhout et al., 2006).

Interestingly, a study on upstream and downstream guppies from a single river showed that MHC diversity did not differ between locations, despite the fact that parasite loads and the selection coefficient were higher in the upstream location (van Oosterhout et al., 2006). In that case, downstream populations were approximately 24 times as large as upstream populations, which may have prevented upstream populations from achieving a greater level of diversity. Our analysis of microsatellites suggests that downstream longnose dace populations did not harbor increased neutral genetic diversity relative to upstream populations, and thus, it is unlikely that directionality is playing a role in MHC variation in our system; instead, factors unrelated to geography are probably the biggest drivers of MHC diversity.

We hypothesized that balancing selection would occur more strongly in some populations than in others due to variation in pathogen susceptibility among populations, or due to other ecological or environmental factors. One type of balancing selection occurs when heterozygotes have higher fitness than homozygotes due to overdominance. Heterozygote advantage should manifest as a higher‐than‐expected frequency of heterozygotes, based on Hardy–Weinberg equilibrium. An unexpected result was that observed heterozygosities at the MHC II β exon 2 were *lower* than expected under neutrality. The larger the number of MHC molecules encoded by different alleles, the greater the probability that a particular antigen will be presented to T cells, and thus the greater the immunological protection. Studies predict, however, that an *optimal* number of MHC molecules exist and that more is not necessarily better (Nowak, Tarczy‐Hornoch, & Austyn, 1992). As intra‐individual MHC diversity increases, so does the potential for mechanisms to prevent autoreactivity, which leads to inactivation of the T‐cell repertoire (Nowak et al., 1992). Results from several studies suggest an intermediate number of MHC alleles may result in highest fitness (e.g., Hablützel et al., 2014; Kalbe et al., 2009; Stiebens, Merino, Chain, & Eizaguirre, 2013). Despite these predictions, we failed to detect any signatures of heterozygote advantage in any population. This result is unlikely to be due to a genetic bottleneck, which typically reduces the number of alleles more rapidly than heterozygosity is reduced (Cornuet & Luikart, 1996).

Another type of balancing selection occurs at the population level to increase the allelic diversity and nucleotide variation. This occurs when rare alleles have higher fitness than common alleles (i.e., negative frequency‐dependent selection) and can result from an arms race between pathogen and host. We were able to test for negative frequency‐dependent selection by inferring an excess of nonsynonymous substitutions, suggesting that nonsynonymous mutations are subject to positive selection, whereas synonymous mutations are often lost due to drift. Most estimates of dN and dS do not accommodate intragenic recombination, which has been shown to shape MHC diversity in a number of systems (e.g., Gómez, Conejeros, Marshall, & Consuegra, 2010; Lam, Shen, Chia, Chan, & Ren, 2013; Zhao et al., 2013). Recombination within a gene can distort any detectable patterns of selection on the gene (Ramírez‐Soriano, Ramos‐Onsins, Rozas, Calafell, & Navarro, 2008; Wilson & McVean, 2006) because analyses are based on correlations between polymorphic sites and nucleotide differences, or synonymous and nonsynonymous sites and mutations, along an *entire* sequence. We therefore based our conclusions on results from combined analyses of dN/dS ratios and intragenic recombination.

Overall, our results did suggest the presence of selection for increased intrapopulation diversity at specific amino acid residues and that more amino acid residues were under selection in upstream populations. The MHC II β exon 2 involves peptide binding; we note (Figure 3) which amino acids were inferred to be the human leukocyte antigen‐binding sites (Brown et al., 1993). Studies have predicted that dN/dS ratios should be higher for the peptide‐binding region than the non‐peptide‐biding region of MHC II β exon 2, but some have found low support for this prediction in cyprinids (e.g., Hedrick, Parker, & Lee, 2001; Ottová et al., 2005). It has thus been suggested that the peptide‐binding regions in fishes may not correspond well to those regions in humans (Ottová et al., 2005). Given the high dissimilarity of our alleles to other DAB3 alleles identified for cyprinids, we refrained from making predictions about peptide‐binding regions in our samples.

We may have expected variation in MHC diversity to occur along a gradient if environmental and ecological variables also vary along a gradient. For example, pollution occurs along a river gradient and impacts ectoparasites, free‐living larval forms, or intermediate hosts (as in Krause, McLaughlin, & Marcogliese, 2010). This prediction was supported by our results—populations from upstream sites showed increased balancing selection. Similarly, gradients in MHC diversity were observed in Atlantic salmon (*Salmo salar*) along a temperature gradient. Increased allelic diversity at the MHC II β peptide‐binding region, but not at the non‐peptide‐binding region, with increased temperatures along a gradient indicates that temperature may be a selective force acting on MHC diversity, as bacterial communities become more diverse with increasing temperature (Dionne, Miller, Dodson, Caron, & Bernatchez, 2007).

Balancing selection on the MHC has been demonstrated in a number of other systems in other contexts (reviews: Eizaguirre & Lenz, 2010; Fijarczyk & Babik, 2015; Piertney & Oliver, 2006; Sommer, 2005). An interesting parallel exists between our study and that of the threespine stickleback from the St. Lawrence River estuary (McCairns, Bourget, & Bernatchez, 2011), which showed evidence of diversifying selection (higher rate of nonsynonymous mutations than synonymous mutations) in freshwater populations, yet not in marine populations (McCairns et al., 2011). This result was found despite a higher diversity of parasite taxa in the marine fish, suggesting that MHC diversity may be related to the specific parasites present and not parasite diversity. That study and ours suggest that balancing selection may be stronger in some environments than in others, potentially due to differences in pathogen communities among environments.

Several studies have shown that, when subjected to environmental stressors such as hypoxia, temperature extremes, or heavy metal contamination, fish infected with pathogens had higher mortality than uninfected fish (reviewed by Marcogliese & Pietrock, 2011). These findings suggest that, had these infected fish possessed MHC variation that caused them to be more susceptible to pathogens, natural selection would have led to a reduction in these maladapted MHC alleles. This might be the case for specific alleles that do not confer immune protection to any pathogens in the environment, or common alleles to which pathogens are evolving resistance. In contrast, studies on the effects of the pathogenic bacterium *Vibrio harveyi* on killifish (*Fundulus heteroclitus*) from a harbor severely contaminated with polychlorinated biphenyls found no increased mortality relative to killifish from a reference population (Nacci et al., 2009). Similarly, the experimental administration of toxaphene to Arctic charr did not increase susceptibility to cestode infection (Blanar, Curtis, & Chan, 2005). These results indicate that immunosuppression leading to reduced resistance or tolerance to pathogens may not always occur for specific contaminants or fish populations.

## 5 Conclusions

We found evidence of balancing selection (increased rate of nonsynonymous mutations, relative to synonymous mutations) at several amino acid residues in the MHC II β in longnose dace. Furthermore, an increased number of amino acid residues were under such selection in upstream sites, relative to downstream sites. Despite this result, we found no evidence of divergent selection between upstream and downstream sites, which would have led to different alleles occurring in different frequencies in upstream versus downstream populations. Although MHC II β diversity was low in our samples, weak variation in microsatellite alleles and a lack of isolation by distance in these samples suggest that equilibrium conditions may not have yet been achieved in this postglacial system. We infer that variation in balancing selection between upstream and downstream populations in this system may be driven by different pathogen communities that are able to thrive under the different environmental conditions present and may have obscured patterns of genetic differentiation. Future work should involve characterizing other immune response genes and loci (e.g., DAB1), characterizing pathogens from different sites, and determining host susceptibility using experimental infections.

## Conflict of Interest

None declared.
