# Supplementary material for: The evolution of the major histocompatibility complex in upstream versus downstream river populations of the longnose dace
Source: Ecol Evol. 2017 Apr 1;7(10):3297–311. doi: 10.1002/ece3.2839 (PMC5433983; doi:10.1002/ece3.2839)
Supplement: Supplementary file 1 [file ECE3-7-3297-s001.docx]

Appendix

Table S1. Coordinates for each of the sites sampled, including the code names that correspond to code names in Figure 1.

| **Site** | **Code** | **River** | **Latitude** | **Longitude** |
| --- | --- | --- | --- | --- |
| McKinnon Flats | BOW6 | Bow | 50.805458 | -113.696329 |
| Carseland Weir | BOW7 | Bow | 50.823518 | -113.442923 |
| Edworthy Park | BOW1 | Bow | 51.064533 | -114.153277 |
| Douglasdale | BOW3 | Bow | 50.936655 | -113.997098 |
| Policemans Flats | BOW4 | Bow | 50.841865 | -113.952096 |
| Cottonwood Golf Course | BOW5 | Bow | 50.853882 | -113.857603 |
| Inglewood | BOW2 | Bow | 51.037735 | -114.010708 |
| Olin Bridge | OLD1 | Oldman | 49.72744 | -114.08622 |
| Highway 36 | OLD8 | Oldman | 49.95958 | -112.08569 |
| Highway 2 | OLD2 | Oldman | 49.3323 | -113.492 |
| Highway 3A | OLD3 | Oldman | 49.791 | -113.124 |
| Popson Park | OLD4 | Oldman | 49.64177 | -112.85566 |
| Pavan Park | OLD5 | Oldman | 49.75254 | -112.85085 |
| Highway 845 | OLD6 | Oldman | 49.85722 | -112.6244 |
| Taber | OLD7 | Oldman | 49.81413 | -112.16817 |
| Highway 62 | MILK1 | North Milk | 49.0936111 | -112.7744444 |
| Weir Bridge | MILK2 | Milk | 49.1038889 | -111.6994444 |
| Peace River Townsite | PEACE1 | Heart | 56.2305556 | -117.285 |
| Whitemud Downstream | PEACE2 | Whitemud | 56.6066667 | -117.2152778 |
| Penhold Bridge | RDR1 | Red Deer | 52.14395 | -113.9679583 |
| Drumhellar | RDR2 | Red Deer | 51.4673667 | -112.71235 |
| Devon | NSR1 | North Saskatchewan | 53.3704222 | -113.7517 |

Table S2. Pairwise distance values calculated from MHC sequences within populations. Φ_ST_ values from Arlequin (110 permutations) on the bottom diagonal (asterisks indicate statistical significance at the α = 0.05 level). Dxy, the average number of nucleotide substitutions per site between populations on the upper diagonal.

|  | Bow Up | Bow Down | Oldman Up | Oldman Down | Milk |
| --- | --- | --- | --- | --- | --- |
| Bow Up | - | 0.01982 | 0.02211 | 0.01997 | 0.02164 |
| Bow Down | -0.02050 | - | 0.02132 | 0.01909 | 0.02051 |
| Oldman Up | -0.01712 | -0.00555 | - | 0.02154 | 0.02269 |
| Oldman Down | -0.01782 | -0.00924 | 0.00001 | - | 0.02092 |
| Milk | 0.06016* | 0.06001* | 0.05012* | 0.07337* | - |

Table S3. Tests for an excess of nonsynonymous mutations. dN/dS were estimated using DnaSP. Z (an estimate of dN-dS) was estimated using MEGA6. dN, dS, and Z were estimated for the coding region only. Significance levels are not available for dN/dS.

| River | Location | dN/dS | Z | P [Z = 0] |
| --- | --- | --- | --- | --- |
| Bow | Up | 3.537 | 0.919 | 0.360 |
| Bow | Down | 2.451 | 0.682 | 0.497 |
| Oldman | Up | \| 3.535 \| \| --- \| | 1.09 | 0.278 |
| Oldman | Down | \| 2.447 \| \| --- \| | 0.708 | 0.481 |
| Milk |  | 2.460 | 0.584 | 0.560 |

Table S4. Summary of ANOVA results comparing allelic richness and gene diversity of *R. cataractae* among sites within five Alberta river systems sampled during August 2010 and 2012.

|  |  | | Allelic richness | | | Gene diversity | | |
| --- | --- | --- | --- | --- | --- | --- | --- | --- |
| River system | |  | F | df | P | F | df | P |
| Bow |  | | 0.0098 | 6, 70 | 1 | 0.1022 | 6, 70 | 0.9959 |
| Oldman |  | | 0.0462 | 7, 80 | 0.9999 | 0.0682 | 7, 80 | 0.9995 |
| Milk |  | | 0.0312 | 1, 20 | 0.8616 | 0.1025 | 1, 20 | 0.7522 |
| Peace |  | | 0.0266 | 1, 20 | 0.8721 | 0.0035 | 1, 20 | 0.9535 |
| Red Deer |  | | 0.025 | 1, 20 | 0.8795 | 0.037 | 1, 20 | 0.9524 |
